# Breakthrough infections after COVID-19 vaccinations do not elicit platelet hyperactivation and are associated with high platelet–lymphocyte and low platelet–neutrophil aggregates

**DOI:** 10.1016/j.rpth.2023.102262

**Published:** 2023-11-14

**Authors:** Francesca Maiorca, Ludovica Lombardi, Ramona Marrapodi, Davide Pallucci, Annamaria Sabetta, Maria Antonella Zingaropoli, Valentina Perri, Davide Flego, Giulio Francesco Romiti, Bernadette Corica, Marzia Miglionico, Gianluca Russo, Patrizia Pasculli, Maria Rosa Ciardi, Claudio M. Mastroianni, Franco Ruberto, Francesco Pugliese, Fabio Pulcinelli, Valeria Raparelli, Roberto Cangemi, Marcella Visentini, Stefania Basili, Lucia Stefanini

**Affiliations:** 1 Department of Translational and Precision Medicine, Sapienza University of Rome, Rome, Italy; 2 Department of Public Health and Infectious Diseases, Sapienza University of Rome, Rome, Italy; 3 Department of Specialist Surgery and Organ Transplantation “Paride Stefanini,” Sapienza University of Rome, Rome, Italy; 4 Department of Experimental Medicine, Sapienza University of Rome, Rome, Italy; 5 Department of Translational Medicine, University of Ferrara, Ferrara, Italy; 6 Faculty of Nursing, University of Alberta, Edmonton, Alberta, Canada; 7 University Center for Studies on Gender Medicine, University of Ferrara, Ferrara, Italy; 8 Istituto Pasteur Italia—Fondazione Cenci Bolognetti, Rome, Italy

**Keywords:** COVID-19, immunothrombosis, platelet aggregates, respiratory distress syndrome, vaccination

## Abstract

**Background:**

Severe COVID-19 is associated with an excessive immunothrombotic response and thromboinflammatory complications. Vaccinations effectively reduce the risk of severe clinical outcomes in patients with COVID-19, but their impact on platelet activation and immunothrombosis during breakthrough infections is not known.

**Objectives:**

To investigate how preemptive vaccinations modify the platelet-immune crosstalk during COVID-19 infections.

**Methods:**

Cross-sectional flow cytometry study of the phenotype and interactions of platelets circulating in vaccinated (*n* = 21) and unvaccinated patients with COVID-19, either admitted to the intensive care unit (ICU, *n* = 36) or not (non-ICU, *n* = 38), in comparison to matched SARS-CoV-2–negative patients (*n* = 48), was performed.

**Results:**

In the circulation of unvaccinated non-ICU patients with COVID-19, we detected hyperactive and hyperresponsive platelets and platelet aggregates with adaptive and innate immune cells. In unvaccinated ICU patients with COVID-19, most of whom had severe acute respiratory distress syndrome, platelets had high P-selectin and phosphatidylserine exposure but low capacity to activate integrin αIIbβ3, dysfunctional mitochondria, and reduced surface glycoproteins. In addition, in the circulation of ICU patients, we detected microthrombi and platelet aggregates with innate, but not with adaptive, immune cells. In vaccinated patients with COVID-19, who had no acute respiratory distress syndrome, platelets had surface receptor levels comparable to those in controls and did not form microthrombi or platelet–granulocyte aggregates but aggregated avidly with adaptive immune cells.

**Conclusion:**

Our study provides evidence that vaccinated patients with COVID-19 are not associated with platelet hyperactivation and are characterized by platelet–leukocyte aggregates that foster immune protection but not excessive immunothrombosis. These findings advocate for the importance of vaccination in preventing severe COVID-19.

## Essentials

• The impact of preemptive vaccinations on immunothrombosis in breakthrough infections is unknown.
• We compared the platelet-immune crosstalk of vaccinated or unvaccinated patients with COVID-19.
• Vaccinated patients did not display hyperactive platelets and platelet–granulocyte aggregates.
• Vaccinations by accelerating virus containment may be limiting excessive immunothrombosis.

## Graphical abstract

**Figure:**
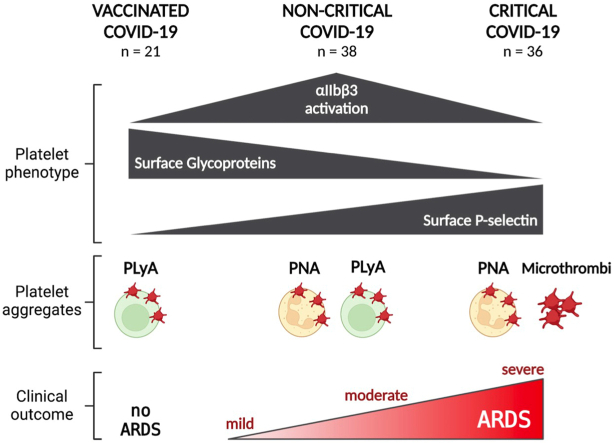

## 1 Introduction

SARS-CoV-2 infections evoke immunothrombosis, a physiological process coordinated by the hemostatic and the immune system to limit the systemic spread of invading pathogens [1]. Excessive immunothrombosis results in thromboinflammatory complications that exacerbate the severity of COVID-19 by reducing gas exchange in the lungs and contributing to multiorgan failure [2,3].

The world-scale deployment of anti–SARS-CoV-2 vaccines has significantly reduced the risk of SARS-CoV-2 infection, hospitalization, severe illness, and death [4]. Despite the rising number of breakthrough infections due to the emergence of novel variants and the waning of immunity, vaccinations have remained effective in reducing the risk of severe clinical outcomes [5,6]. Hence, we investigated if preemptive vaccinations modify the platelet-immune crosstalk and the immunothrombotic response during COVID-19 infections.

## 2 Methods

### 2.1 Study population

Ninety-five SARS-CoV-2–positive patients (aged >18 years), admitted at the Umberto I Hospital (Rome, Italy) from September 2020 to December 2021, were included in the study. SARS-CoV-2 infection diagnosis was made by COVID-19 real-time reverse-transcriptase polymerase chain reaction test on nasopharyngeal swab. All study patients were Caucasians and were enrolled within 72 hours of hospital or intensive care unit (ICU) admission, after first-line treatment had been administered. Standard treatment included remdesivir for 3 days and prophylactic dosage of enoxaparin and dexamethasone for patients undergoing oxygen therapy. Twenty-one patients with COVID-19, enrolled between October and December 2021, had received a complete course of vaccination more than 2 weeks prior to infection. Patients with an incomplete course of vaccination were not included in the study. Forty-eight SARS-CoV-2–negative patients were recruited as controls from the internal medicine ward to match the age and comorbidities of the patients with COVID-19. All participants gave written informed consent. The study was approved by the Ethics Committee of our institution (EC identifier: 5870; ClinicalTrials.gov identifier: NCT04497402).

### 2.2 Platelet phenotypic and functional analysis

Platelet analyses were performed within 30 minutes from blood withdrawal as described previously [7]. Briefly, citrated whole blood diluted 1:10 with Tyrode’s buffer was incubated with saturating concentrations of α-CD41-APC, α-GPVI-PE, α-CD42b-PE, or α-CD31-FITC (BD Bioscience), and the expression of each receptor was quantified based on the median fluorescence intensity among CD41^+^ events. To monitor platelet basal activation and responsiveness, diluted blood was labeled with α-CD62P-PE and PAC1-FITC (BD Bioscience) in the absence or presence of 0.5-μM adenosine diphosphate (ADP) or 10-ng/mL convulxin (CVX). To detect procoagulant platelets, whole blood diluted with Tyrode’s buffer containing 2-mM Ca^2+^ and Gly-Pro-Arg-Pro (fibrin polymerization inhibitor) was incubated with α-CD41-APC and Annexin V-PE (Sony Biotechnology). All samples were fixed with 1% formaldehyde in phosphate-buffered saline, acquired on a BD Accuri C6 Plus, and analyzed with the FlowJo LLC software.

### 2.3 Analysis of the inner mitochondrial membrane integrity

The integrity of the inner mitochondrial membrane was assessed by costaining washed platelets (3 × 10^7^ cells/mL) with 2 cell-permeant mitochondrial dyes: MitoTracker Green FM (Invitrogen, M7514), which accumulates in mitochondria independently of the inner membrane potential (proportional to mitochondrial size), and MitoTracker CMXRos (Invitrogen, M7512), which is retained only in active mitochondria with an impermeable inner membrane.

After incubation of the 2 probes (2 μM) with washed platelets for 20 minutes in the absence or presence of 10 ng/mL CVX, samples were diluted with phosphate-buffered saline, acquired immediately on a BD Accuri C6 Plus, and analyzed with the FlowJo LLC software. The ratio of the fluorescence emitted by the 2 dyes was used to quantify the integrity of the inner mitochondrial membrane.

### 2.4 Platelet–leukocyte and platelet–platelet aggregate quantification

Within 15 minutes from blood withdrawal, 50 μL of whole blood were incubated with fluorochrome-conjugated monoclonal antibodies α-CD66b-PE, α-CD56-PEDazzle594, α-CD19-PerCPCy5.5, α-CD16-PECy7, α-CD14-APC, α-CD4-Alexa700, α-CD25-Bv421, α-CD3 Bv510, α-CD8 Bv605 (SONY Biotechnology), and α-CD41-BB515 (BD Bioscience). After 15 minutes of incubation, red cell lysis was performed with BD FACS Lysing Solution. Flow cytometry acquisition was conducted on a BD LSRFortessa and analyzed with FlowJo. At least 50,000 events in the singlet gate were acquired. Fluorescence-minus-one controls were performed to ensure proper gating. Platelet–leukocyte aggregates (PLAs) were identified based on the expression of CD41a in the individual leukocyte subpopulations (Supplementary Figure S1). The effect of blocking P-selectin on PLAs was assessed by preincubating whole blood with the α-P-selectin blocking antibody (clone AK4) for 15 minutes prior to staining.

Circulating microthrombi were identified by gating for CD41a+ events larger than individual platelets (Forward Side Scatter High [FSC^high^]) and then by excluding events positive for T cell–specific, B cell–specific, neutrophil-specific, and monocyte-specific markers (Supplementary Figure S2).

### 2.5 Characterization of platelet-bound and -unbound circulating B cells

The phenotype of circulating B cells was assessed by flow cytometry with an antibody panel including α-CD19-PerCPCy5.5 and α-CD41-APC to discriminate platelet-bound and -unbound B cells and leukocyte markers of activation (α-CD69-FITC) and differentiation (α-CD62L-PE). Fifty microliters of whole blood were incubated for 15 minutes with saturating concentrations of the antibodies and then for 10 minutes with the BD FACS Lysing Solution. Flow cytometry acquisition was conducted on a BD Accuri C6 Plus and analyzed with FlowJo software.

### 2.6 Quantification of cell-free DNA

Cell-free double-stranded DNA (dsDNA) was quantified in the serum of the patients using the Quant-iT PicoGreen dsDNA Assay-Kit (Thermo Fisher Scientific) according to the manufacturer’s instructions. Samples were diluted 1:20 in Tris-EDTA buffer, and Quant-iT PicoGreen reagent was added in a 1:1 ratio. After 5 minutes in the dark, the fluorescence was read in a VICTOR3 plate reader (Perkin Elmer). The concentration of the circulating cell-free dsDNA was calculated using the kit standards.

### 2.7 Statistical analysis

Categorical variables are expressed as counts and percentages. Continuous variables are reported as mean ± SD or median and IQR. Normally distributed continuous variables were compared using Student’s *t*-tests, and nonnormally distributed ones were compared with Mann–Whitney U-test. Appropriate 2-tailed nonparametric tests were used to evaluate intergroup differences (Kruskal–Wallis test). Correlations were assessed by simple regression analysis. A 2-sided *P* value of <.05 was considered statistically significant. All analyses were performed using GraphPad Prism 9.

## 3 Results

### 3.1 Disease severity and vaccination status are not associated with significant changes in the platelet count of patients with COVID-19

**Figure 1:**
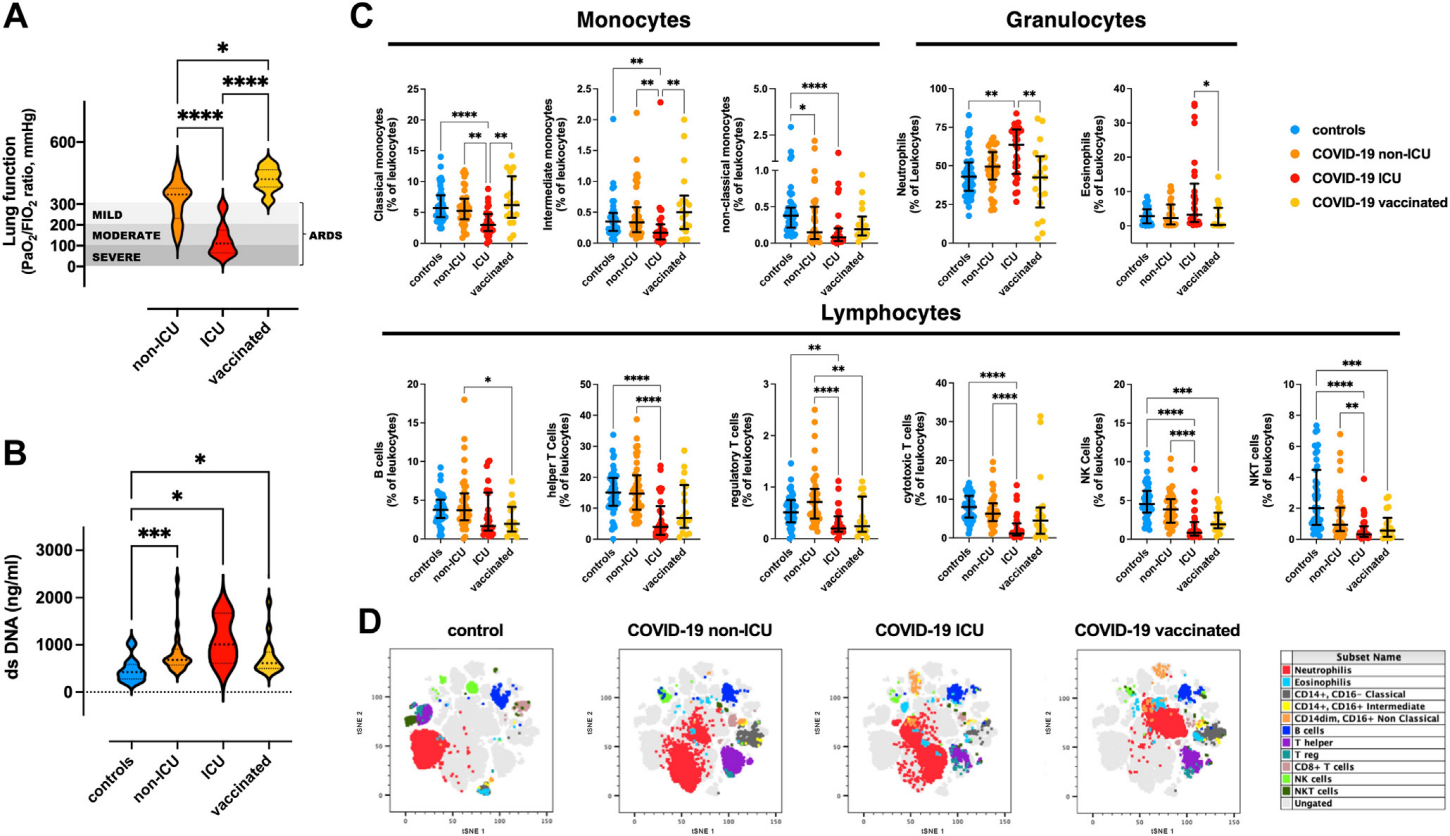
Clinical, serological, and immunological markers of disease severity in vaccinated and unvaccinated patients with COVID-19. (A) Violin plot of the PaO_2_/FiO_2_ ratio of vaccinated (yellow) and unvaccinated patients with COVID-19, admitted to the intensive care unit (ICU, red) or not (non-ICU, orange). The median is shown as a black dotted line, and the upper and lower quartiles are indicated by grey dotted lines. The PaO_2_/FiO_2_ ratio is a clinical measure of lung function that is used to stratify patients with severe (<100 mmHg, dark grey area), moderate (100-200 mmHg, medium grey area), and mild (200-300 mmHg, light grey area) acute respiratory distress syndrome (ARDS). Kruskal–Wallis test and Dunn’s multiple comparison test were used for intergroup analysis. (B) Violin plot of the circulating cell-free double-stranded DNA (dsDNA) in the serum of non-ICU (orange), ICU (red), and vaccinated (yellow) patients with COVID-19 and controls (blue). Kruskal–Wallis test and Dunn’s multiple comparison test were used for intergroup analysis. (C) Immunophenotype of non-ICU, ICU vaccinated COVID-19, and control patients. Leukocyte subpopulations were identified by multiparameter flow cytometry on a BD LSRFortessa and analyzed with the FlowJo LLC software, version 10.8.1, based on the expression of surface markers: classical (CD14^+^CD16^−^), intermediate (CD14^+^CD16^+^), and nonclassical (CD14^dim^CD16^+^) monocytes; neutrophils (CD66^+^CD16^+^); eosinophils (CD66^+^CD16^−^); B cells (CD3^−^CD19^+^); CD4^+^ (CD3^+^CD4^+^), regulatory (CD3^+^CD4^+^CD25^high^), and CD8^+^ (CD3^+^CD8^+^) T cells, and natural killer (NK, CD3^−^CD56^+^) and natural killer T cells (NKT, CD56^+^CD3^+^). Shown are the median ± IQR of the percentage of each subset relative to all leukocytes. Kruskal-Wallis test and Dunn’s multiple comparison test were used for intergroup analysis. ∗*P* < .05; ∗∗*P* < .01; ∗∗∗*P* < .001; ∗∗∗∗*P* < .0001. (D) Representative *t*-distributed stochastic neighbor embedding (t-SNE) dot plot of 4 patients, 1 for each studied group, analyzed on the same day. The t-SNE plot was obtained by combining the data of the 4 patients before identifying the leukocyte subsets, which are labeled with different colors as shown in the legend.

A total of 95 SARS-CoV-2–positive hospitalized patients (62% males; 64 ± 17 years) were enrolled in this study and compared to 48 SARS-CoV-2–negative patients (58% males; 57 ± 19 years) matched for age and comorbidities. Patients with COVID-19 were stratified based on disease severity and vaccination status. Clinical and demographic characteristics of each group are summarized in the Table. Twenty-one of the patients with COVID-19 had received a complete course of vaccination more than 2 weeks prior to infection (vaccinated). Among unvaccinated patients with COVID-19, 36 required mechanical ventilation and were admitted to the ICU and 38 were not critical (non-ICU). Among the vaccinated patients with COVID-19 only 3 were admitted to the ICU and all of them were males, older than 70 years of age and with type 2 diabetes. Groups were well matched by age, but there was a prevalence of male patients in the ICU group, which reflects well-established sex differences in COVID-19 incidence [8]. Hospitalized patients with COVID-19 had comorbidities consistent with those reported in previously published studies, including hypertension, diabetes, and a history of cardiovascular disease. Most of the ICU patients experienced severe acute respiratory distress syndrome (ARDS) (PaO_2_/FiO_2_ ratio: 114 [69-177] mmHg) (Figure 1A). Non-ICU patients were more heterogeneous in terms of lung function (PaO_2_/FiO_2_ ratio: 350 [234-380] mmHg). Vaccinated patients with COVID-19 did not experience pulmonary complications (PaO_2_/FiO_2_ ratio: 424 [386-470] mmHg). Serum cell-free dsDNA, which is a marker of tissue damage and extracellular traps released by activated neutrophils (NETs), was significantly increased, compared to controls, in all patients with COVID-19, independently of the vaccination status, and was highest in critical patients admitted to the ICU (controls: 423 ng/mL [278-582]; non-ICU: 680 ng/mL [574-909]; ICU: 1009 ng/mL [609-1676]; vaccinated: 615 ng/mL [499-845]) (Figure 1B).

**Table:** Baseline characteristics of the enrolled patients.

Study Groups	Controls	Unvaccinated Non-ICU COVID-19	Unvaccinated ICU COVID-19	Vaccinated COVID-19
Enrolled, *n*	48	38	36	21
Age (y), mean ± SD	57 ± 19	57 ± 21	66 ± 11	69 ± 17
SARS-CoV-2 RT-PCR	Negative	Positive	Positive	Positive
Sex, % males	58	48	79	61
Ethnicity, % White	100	100	100	100
Cardiovascular disease, %	26	30	46	24
Diabetes, %	21	15	38	26
Hypertension, %	34	39	46	35
Platelets (×10^3^/μL), median (IQR)	218 (191-273)	251 (182-338)	208 (118-262)	260 (204-413)

ICU, intensive care unit; RT-PCR, reverse-transcription polymerase chain reaction.

In line with previous reports [9,10], disease severity was not accompanied by changes of the platelet count (Table) but associated with marked reductions in the frequency of circulating lymphocytes and monocytes and a significant increase in the frequency of neutrophils (Figure 1C, D) [11]. Vaccinated patients with COVID-19 displayed a normal frequency of platelets, granulocytes, and monocytes but had a relatively low frequency of lymphocytes in peripheral venous blood.

### 3.2 Changes in the expression level of platelet surface receptors of unvaccinated, but not of vaccinated, patients with COVID-19

**Figure 2:**
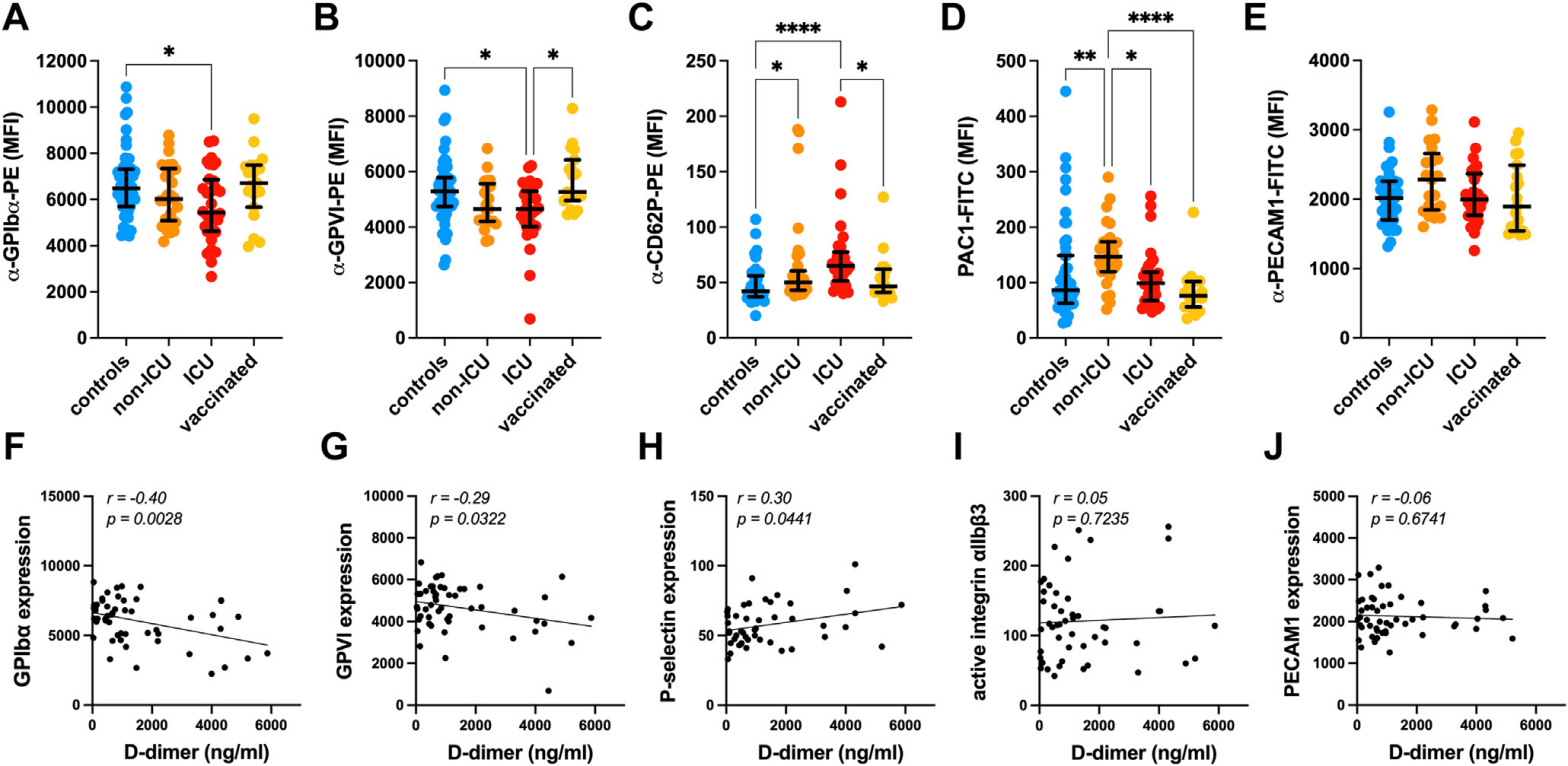
Platelet surface receptor expression of vaccinated and unvaccinated patients with COVID-19 in relation to their D-dimer levels. Surface expression of platelet receptors of SAR-CoV-2–negative control patients (blue, controls), unvaccinated patients with COVID-19 admitted to the intensive care unit (ICU, red) or not (non-ICU, orange), and vaccinated patients with COVID-19 (yellow, vaccinated) detected by flow cytometry after staining of citrated (nonstimulated) whole blood with (A) α-CD42b-PE (GPIbα), (B) α-GPVI-PE, (C) α-CD62P-PE (P-selectin), (D) PAC1-FITC (active αIIbβ3), and (E) α-CD31-FITC (PECAM1). Samples were acquired on a BD Accuri C6 Plus and analyzed with the FlowJo LLC software, version 10.8.1. Graphs show the median fluorescence intensity (MFI) ± IQR. Kruskal–Wallis test and Dunn’s multiple comparison test were used for intergroup analysis. ∗*P* < .05; ∗∗*P* < .01; ∗∗∗∗*P* < .0001. Simple linear regression analysis between the surface receptor expression of (F) GPIbα, (G) GPVI, (H) P-selectin, (I) active αIIbβ3, and (J) PECAM1 with the D-dimer concentration expressed in nanograms per milliliter. On the right top corner of each graph are shown the correlation coefficient (*r*) and the *P* value.

To assess whether disease severity or vaccination status affected the platelet phenotype, we studied the surface expression level of some platelet receptors by flow cytometry. The most distinctive feature of circulating platelets in patients with COVID-19 was the decrease of surface glycoproteins (Figure 2A, B). Unvaccinated ICU patients displayed 15% less GPIbα and GPVI. Unvaccinated non-ICU patients had an intermediate phenotype between ICU and matched control patients. P-selectin (CD62P) exposure on the plasma membrane, a marker of α-granule secretion, increased with severity in unvaccinated patients with COVID-19 (Figure 2C). Binding of PAC-1, the antibody that detects the active conformation of the integrin receptor αIIbβ3, was increased in non-ICU patients but not in ICU patients (Figure 2D). The surface expression of PECAM-1, an inhibitory adhesion receptor that is cleaved from the leukocyte surface in inflammatory conditions [12,13], was not significantly changed in patients with COVID-19 (Figure 2E). Simple linear regression analysis identified statistically significant negative correlations between the plasmatic D-dimer concentrations and the surface expression of GPIbα (*r* = −0.40, *P* = .0028) and GPVI (*r* = −0.29, *P* = .0322) (Figure 2F, G) and a positive correlation with the surface levels of P-selectin (*r* = +0.30, *P* = .0441) (Figure 2H), but not with the levels of active αIIbβ3 (*r* = +0.05, *P* = .7235) and PECAM-1 (*r* = −0.06, *P* = .6741) (Figure 2I, J). Vaccinated patients with COVID-19 did not show any significant changes in the expression level of these receptors compared to controls.

### 3.3 Platelet responsiveness to agonists negatively correlates with COVID-19 severity

**Figure 3:**
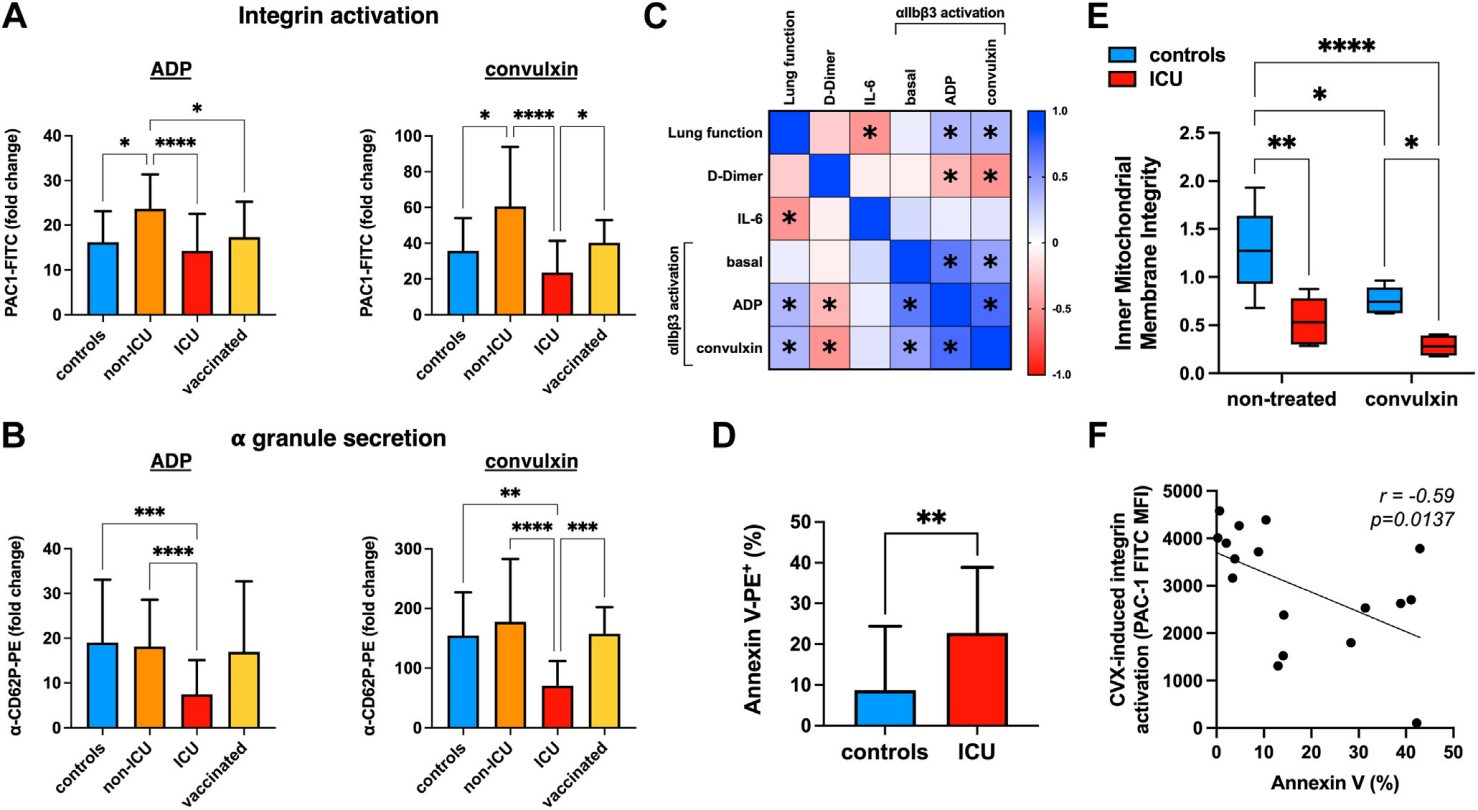
Platelet responsiveness of vaccinated and unvaccinated patients with COVID-19 in relation to their lung function and D-dimer and interleukin 6 (IL-6) levels. Fold change of the (A) activation of integrin αIIbβ3 (PAC1-FITC binding) and of (B) α-granule secretion (α-CD62P-PE binding) in platelets stimulated with adenosine diphosphate (ADP) or convulxin (CVX) (GPVI agonist) relative to the unstimulated platelets from SAR-CoV-2–negative control patients (blue, controls), unvaccinated patients with COVID-19 admitted to the intensive care unit (ICU, red) or not (non-ICU, orange), and vaccinated patients with COVID-19 (yellow, vaccinated). Kruskal–Wallis test with Dunn’s multiple comparison test were used for intergroup analysis. (C) Spearman rank correlation analysis between integrin αIIbβ3 activation in nonstimulated (basal) and ADP- or CVX-stimulated platelets and the lung function (PaO_2_/FiO_2_ ratio), the plasmatic concentration of D-dimer, and that of IL-6. The color of each box indicates the correlation coefficient (*r*), that ranges from +1 (positive correlation, blue) to −1 (negative correlation, red), and the asterisks indicate significant (*P* < .05) correlations. (D) Phosphatidylserine exposure (expressed as percentage of platelets binding to Annexin V-PE) on the outer leaflet of platelets from SAR-CoV-2–negative controls (blue) and ICU COVID-19 patients (red), analyzed by Mann–Whitney U-test. (E) Flow cytometric analysis of the inner mitochondrial membrane integrity quantified as the ratio of the median fluorescence intensity emitted by MitoTracker Red CMXRos and MitoTracker Green FM incubated with nontreated and CVX-stimulated platelets of SAR-CoV-2–negative controls (blue) and ICU patients with COVID-19 (red). Statistical significance was computed by 2-way analysis of variance and Sidak’s multiple comparison test. (F) Simple linear regression analysis between the platelet responsiveness to CVX (CVX-induced integrin activation) and phosphatidylserine exposure (Annexin V binding). On the top right corner, the correlation coefficient (*r*) and the *P* value are shown. ∗*P* < .05; ∗∗*P* < .01; ∗∗∗*P* < .001; ∗∗∗∗*P* < .0001.

To test if circulating platelets of patients with COVID-19 had a different responsiveness to agonists, we stimulated the platelets directly in whole blood and measured their ability to increase P-selectin exposure and integrin αIIbβ3 activation relative to the nonstimulated conditions (Figure 3A, B). Platelets from unvaccinated non-ICU patients activated αIIbβ3 significantly more than controls in response to either weak (ADP) or strong (CVX) agonists, while vaccinated subjects from the same clinical ward had normoresponsive platelets. Platelets from ICU patients, compared to non-ICU patients, had a significantly reduced capacity to activate αIIbβ3 in response to CVX, and an impaired α-granule secretion in response to both ADP and CVX. Platelet responsiveness to agonists negatively correlated with the plasmatic D-dimer concentration (ADP: *r* = −0.34, *P* = .017; CVX: *r* = −0.50, *P* < .001) and positively correlated with the PaO_2_/FiO_2_ ratio (ADP: *r* = +0.36, *P* = .002; CVX: *r* = +0.35, *P* = .002), that is, patients with more severe disease (worse lung function and hypercoagulability) displayed less responsive platelets (Figure 3C). The plasmatic concentration of interleukin 6, which negatively correlated with the PaO_2_/FiO_2_ ratio (*r* = −0.50, *P* = .003), was not related to platelet responsiveness.

To assess whether the reduced integrin activation, we observed in ICU patients with COVID-19 was due to epitope masking by von Willebrand factor or other multimeric proteins, we compared the agonist-induced PAC-1 binding of 3 severe patients and 3 controls in diluted whole blood, platelet-rich plasma, or after platelet isolation. We observed that platelets from patients with COVID-19 were also less responsive after removing other blood cells and plasma (Supplementary Figure S3), suggesting that the hyporesponsiveness was a platelet intrinsic feature. Integrin inactivation occurs in platelets that are turning procoagulant, and it is regulated either by calpain or through a pathway involving the loss of the inner mitochondrial membrane integrity [14]. In ICU patients with COVID-19, we measured an increased percentage of procoagulant platelets (*n* = 17, 22.8 ± 16.1%), identified with the phosphatidylserine-binding protein Annexin V, compared to controls (*n* = 13, 8.7 ± 15.7%) (Figure 3D). We did not detect calpain cleavage of the cytoplasmic tail of the β3 subunit or of the integrin-activating protein talin-1 (data not shown), but we measured a reduced mitochondrial membrane integrity in platelets from ICU patients compared to controls (Figure 3E). Notably, agonist-induced integrin activation was inversely related to Annexin V binding (*r* = −0.59, *P* = .0137) (Figure 3F).

### 3.4 Circulating microthrombi correlate with disease severity and are not detected in vaccinated patients with COVID-19

**Figure 4:**
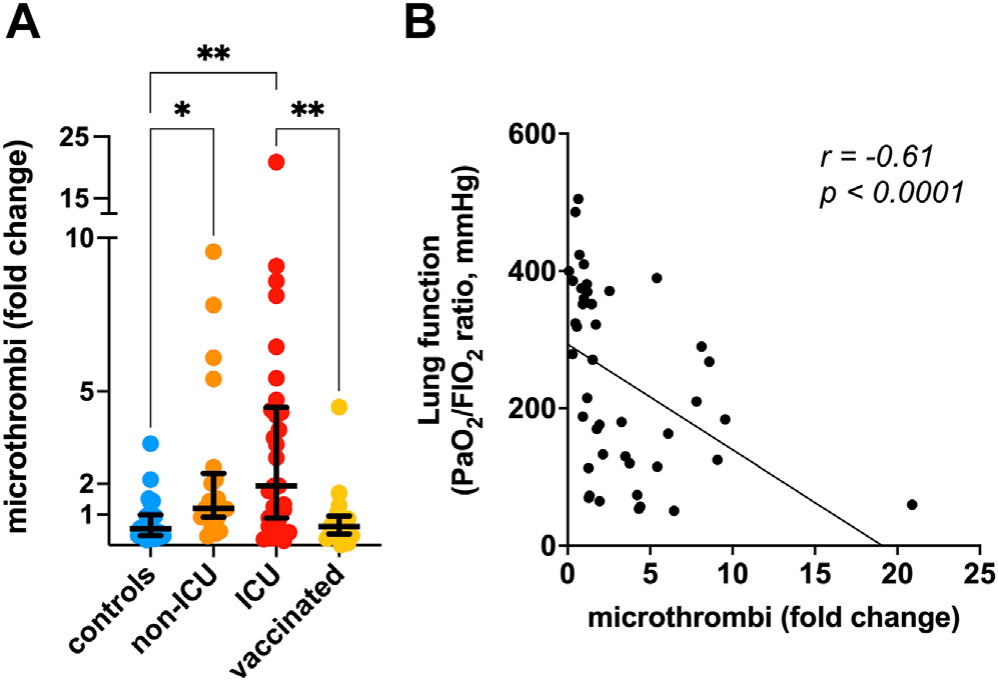
Platelet–platelet aggregates in vaccinated and unvaccinated patients with COVID-19. (A) Bar graph of the relative frequency of circulating platelet–platelet aggregates (microthrombi). Shown is the mean ± SD of the fold change relative to the negative control. Kruskal–Wallis test and Dunn’s multiple comparison test were used for intergroup analysis. ∗*P* < .05; ∗∗*P* < .01. (B) Simple linear regression analysis between the lung function (PaO_2_/FiO_2_ ratio) of the patients and the fold change of circulating microthrombi. On the top right corner, the correlation coefficient (*r*) and the *P* value are shown. ICU, intensive care unit.

To determine if the circulating platelets of patients with COVID-19 could form pathological platelet–platelet aggregates in vivo, we quantified directly in whole blood the CD41a+ events that were larger than regular platelets but did not bind leukocytes, previously described as circulating microthrombi [15,16]. Platelet–platelet aggregates were detected in the blood of unvaccinated COVID-19, most frequently in patients admitted to the ICU, but not in vaccinated patients with COVID-19 (fold change non-ICU: 2.3 ± 2.6; ICU: 3.5 ± 4.1; vaccinated: 0.9 ± 1.0) (Figure 4A). The lung function of patients negatively correlated with the frequency of circulating microthrombi (*r* = −0.61, *P* < .0001), that is, the presence of high levels of microthrombi in circulation was associated with more severe ARDS (Figure 4B).

### 3.5 Distinct platelet–leukocyte interactions in patients with COVID-19 with different disease severity and vaccination status

**Figure 5:**
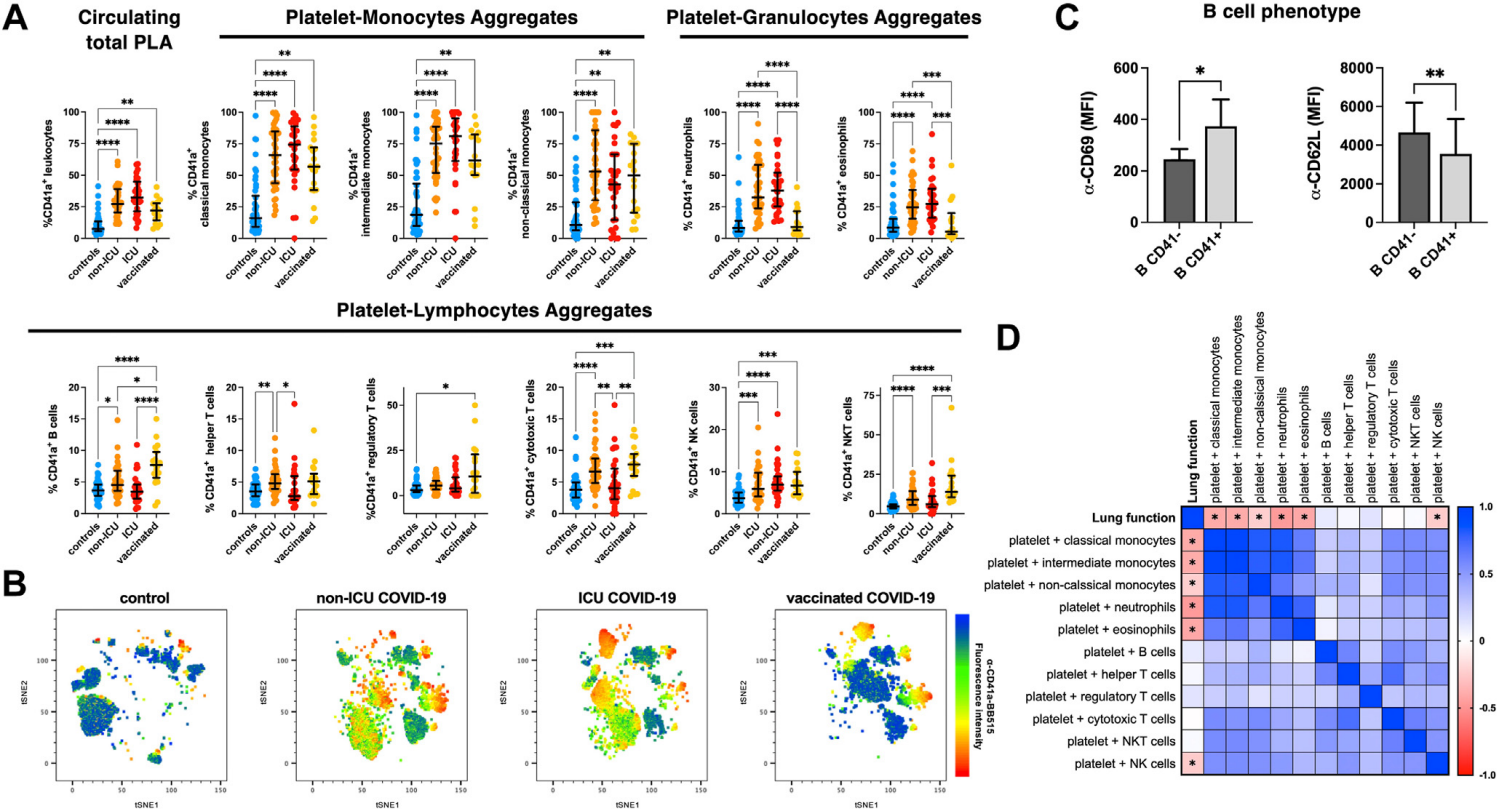
Platelet–leukocyte aggregates (PLAs) in vaccinated and unvaccinated patients with COVID-19. (A) Relative frequencies of the circulating platelet aggregates with neutrophils (CD66^+^CD16^+^); eosinophils (CD66^+^CD16^−^); classical (CD14^+^CD16^−^), intermediate (CD14^+^CD16^+^), and nonclassical (CD14^dim^CD16^+^) monocytes; B cells (CD3^−^CD19^+^); helper (CD3^+^CD4^+^), cytotoxic (CD3^+^CD8^+^), and regulatory (CD3^+^CD4^+^CD25^high^) T cells; and natural killer (NK, CD3^−^CD56^+^) and natural killer T cells (NKT, CD56^+^CD3^+^). Flow cytometry acquisition was performed on a BD LSRFortessa and analyzed with the FlowJo LLC software. PLAs were identified based on the expression of CD41a in the individual leukocyte subpopulations and shown is the median ± IQR of the percentage of CD41a^+^ leukocytes of different subsets. Kruskal–Wallis test and Dunn’s multiple comparison test were used for intergroup analysis. (B) *T*-distributed stochastic neighbor embedding (t-SNE) plot showing the CD41a^+^ leukocytes in 4 representative patients. Red dots indicate higher CD41a expression. (C) B cell phenotype in vaccinated patients with COVID-19. Bar graphs of the expression (median fluorescence intensity [MFI]) of CD69 (activation marker) and CD62L (L-selectin, migration marker) on platelet-bound (B CD41^+^) and platelet-unbound (B CD41^−^) B cells. Statistical significance was determined by paired *t*-test analysis. ∗*P* < .05; ∗∗*P* < .01; ∗∗∗*P* < .001; ∗∗∗∗*P* < .0001. (D) Spearman rank correlation analysis between the lung function (PaO_2_/FiO_2_ ratio) and the frequency of PLAs. The color of each box indicates the correlation coefficient (*r*) that ranges from +1 (positive correlation, blue) to −1 (negative correlation, red), and the asterisks indicate significant (*P* < .05) correlations. ICU, intensive care unit.

To study if the different functional and adhesive properties of platelets among the 4 patient groups might influence their ability to interact with circulating immune cells, we stained fresh whole blood with a combination of fluorochrome-tagged antibodies that identify platelets bound to 11 leukocyte subsets [7]. We observed that SARS-CoV-2–positive patients had elevated levels of PLAs independently of their disease severity and vaccination status, but that the frequency of platelet aggregates with specific leukocyte subsets changed across different groups (Figure 5A, B). Among unvaccinated patients with COVID-19, aggregates with innate immune cells accounted for most of the interactions, with non-ICU patients displaying 65 ± 24% of monocytes and 40 ± 21% of neutrophils bound to platelets and ICU patients having 68 ± 26% of monocytes and 40 ± 20% of neutrophils bound to platelets. Conversely, the binding of platelets to lymphocytes, specifically with B cells, helper and cytotoxic T cells, and natural killer T cells, was significantly increased in non-ICU patients but dropped in ICU patients.

Vaccinated patients with COVID-19 displayed a platelet–leukocyte binding profile that was distinct from both ICU and non-ICU unvaccinated patients. These patients had 2 striking features. First, in vaccinated patients with COVID-19, while platelet–monocyte aggregates remained high, we observed a complete normalization of the frequency of platelet–neutrophil and platelet–eosinophil aggregates. Second, in the blood of vaccinated patients with COVID-19 we detected significantly higher levels of circulating platelet–lymphocyte aggregates compared to the other 3 cohorts. Platelets from vaccinated subjects bound most avidly to B cells, regulatory and cytotoxic T cells, and natural killer T cells. The platelet-bound B cells (CD41^+^ B cells) had a distinct phenotype compared to unbound B cells (CD41^−^ B cells) as they expressed higher levels of the activation marker CD69 and lower levels of the adhesion receptor L-selectin (CD62L) (Figure 5C), which decreases as B cells differentiate into antibody-secreting cells [17].

The lung function of patients negatively correlated with the frequency of platelet aggregates with granulocytes (neutrophils: *r* = −0.47, *P* < .0001; eosinophils: *r* = −0.41, *P* < .0001) and monocytes (classical monocytes: *r* = −0.40, *P* < .0001; intermediate monocytes: *r* = −0.39, *P* < .0001; nonclassical monocytes: *r* = −0.23, *P* = .03), but not with the frequency of platelet–lymphocyte aggregates (PaO_2_/FiO_2_ ratio correlation with platelet–B cell aggregates: *r* = 0.11, *P* = .33), that is, severe ARDS associated with the presence in circulation of high levels of platelet complexes with innate but not with adaptive immune cells (Figure 5D).

### 3.6 *Ex vivo* P-selectin inhibition reduces platelet interactions with innate immune cells while minimally affecting platelet binding to lymphocytes

**Figure 6:**
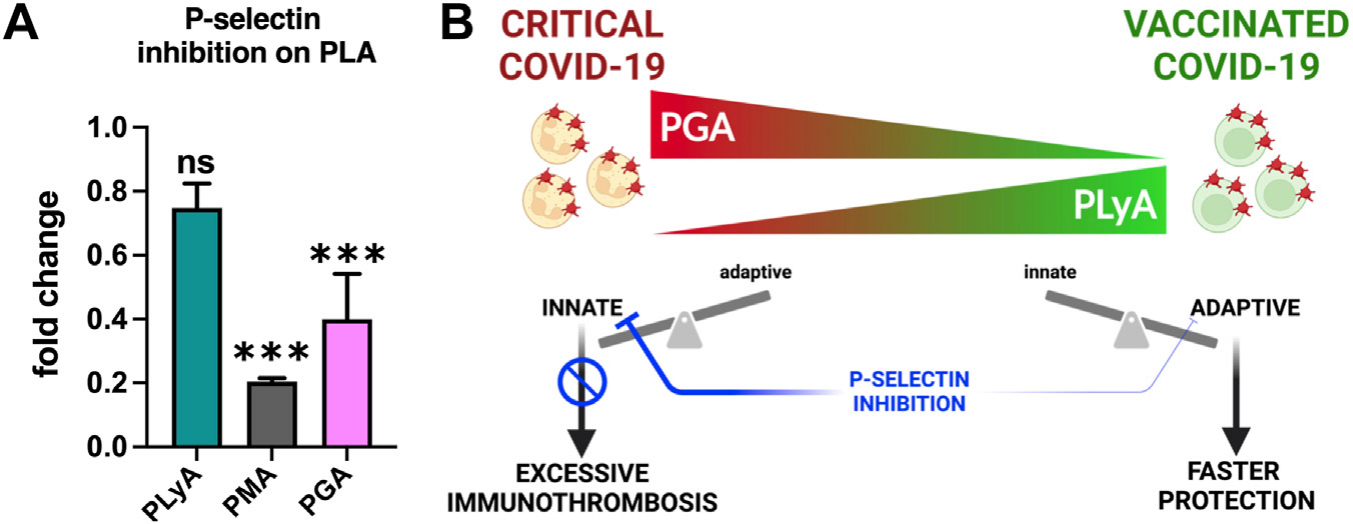
Effect of P-selectin inhibition on the frequency of platelet–leukocyte aggregates (PLAs) in unvaccinated patients with COVID-19. (A) Fold change of the frequency of platelet–lymphocyte aggregates (PLyAs), platelet–monocyte aggregate (PMAs), and platelet–granulocyte aggregates (PGAs) after incubation of whole blood of non–intensive care unit patients with COVID-19 (*n* = 3) with a blocking anti-CD62P antibody (clone AK4). Kruskal–Wallis test and Dunn’s multiple comparison test were used for intergroup analysis. ∗∗∗*P* < .001. (B) Data interpretation model. Intensive care unit and vaccinated patients represent 2 extremes of the COVID-19 spectrum. High PGAs and low PLyAs identify patients in critical conditions that fail to evoke an effective adaptive immune response and compensate with an excessive innate immune response that injures the host and contributes to the pathogenicity of the disease. Low PGAs and high PLyAs characterize vaccinated subjects that by evoking a rapid adaptive immune response are protected from the virus itself and from the pathogenic effects of a prolonged innate immune response. A P-selectin inhibitor would in principle be beneficial to reduce COVID-19 severity by reducing the pathogenic effects of innate immunity while minimally affecting the adaptive immune response. ns, nonsignificant.

Inhibition of P-selectin is being tested to prevent the detrimental effects of immunothrombosis in various settings, including in patients with COVID-19 [18,19]. We tested the effects of an anti–P-selectin blocking antibody on the frequency of the PLAs in the blood of 3 patients with COVID-19 in noncritical conditions. The blocking antibody significantly reduced platelet binding with monocytes and granulocytes, by 80% and 60% respectively, but not with lymphocytes (Figure 6A). Thus, a P-selectin inhibitor would in principle be beneficial to reduce the risk of severe COVID-19 by reducing the pathogenic effects of innate immunity while minimally affecting the adaptive immune response (Figure 6B).

## 4 Discussion

The main finding of our study is that the platelet phenotype and the platelet aggregates implicated in immunothrombosis are modified not only by the severity of the disease but also by the vaccination status of patients with COVID-19.

As others before us, we provide evidence that during SARS-Cov-2 infections platelets become hyperactivated [9,20]. In non-ICU unvaccinated patients, we observed active integrin αIIbβ3 and granule-derived P-selectin exposed on the surface of nonstimulated platelets and increased αIIbβ3 activation after agonist stimulation. In ICU patients, we detected even higher levels of surface P-selectin, reduced surface glycoproteins, increased phosphatidylserine exposure, and impaired ability of platelets to undergo further granule secretion and αIIbβ3 activation upon agonist stimulation. While P-selectin exposure supports platelet binding to active endothelial and immune cells, αIIbβ3 inactivation [14], and glycoprotein shedding [21] combined with increased phosphatidylserine exposure enhance binding of coagulation factors to the platelet surface. Thus, in patients with severe COVID-19, hyperactivated platelets eventually shift from a proaggregatory to a proinflammatory and procoagulant phenotype. Despite the ongoing infection, vaccinated patients are at the opposite end of the platelet activation spectrum as they display normoresponsive platelets with surface levels of GPIbα, GPVI, P-selectin, and active αIIbβ3 comparable to controls.

An elegant study by Schrottmaier et al. [22] has shown that platelet hyporesponsiveness aggravates over time in patients with severe COVID-19 and that it is at least in part due to a factor present in plasma. In our experimental settings the impaired αIIbβ3 activation persisted after removal of the plasma as if the platelets were changed irreversibly by the infection. Although SARS-CoV-2 RNA in not always measurable in serum [23,24], virus debris has been detected inside platelets from patients and has been associated with cell death [25] and to partial desensitization of αIIbβ3 [26]. Thus, one could speculate that when the virus becomes systemic, it can affect platelet function directly.

Viral infections can damage mitochondria [27] and cause premature cellular senescence [28] that is a critical driver of COVID-19 severity. Others have reported mitochondrial dysfunction in platelets of patients with COVID-19 [29], and we show a significant reduction in the inner mitochondrial membrane integrity in platelets of ICU patients, suggesting that mitochondrial dysfunction and the consequent energetic exhaustion may, at least in part, underlie the functional platelet exhaustion of severely infected individuals. Mitochondrial dysfunction is a feature of platelets during aging [30] and may explain the susceptibility of older adults to severe COVID-19.

Notably, platelet responsiveness to agonists positively correlated with the PaO_2_/FiO_2_ ratio, that is, patients with worse lung function displayed less responsive platelets (Figure 3C). Hypoxia can lead to the overproduction of reactive oxygen species and calcium overload in the mitochondria [31] and may be implicated with the impaired platelet reactivity [32] that we see in patients with COVID-19 with severe, but not mild, ARDS.

Platelet hyporesponsiveness, increased Annexin V binding [33], and shedding of GPIbα and GPVI [34] have also been documented in major trauma patients as a result of the direct interaction between platelets and histones released from injured tissues [33,35]. In the serum of ICU patients, we measured significantly higher levels of dsDNA, and we can assume that histones and other danger-associated molecular patterns were also released by activated neutrophils and infection-damaged tissue. Thus, a combination of factors including the systemic spreading of the virus, hypoxia, cell-free histones, and other danger-associated molecular patterns released during the infection [36] contribute to the dysfunctional phenotype of platelets in patients with COVID-19.

Disease severity among unvaccinated patents with COVID-19 was associated with an elevated frequency of circulating FSC^high^CD41^+^ events. Since our flow cytometry panel did not include a DNA marker, we cannot exclude that the FSC^high^CD41^+^ events, which we identified as microthrombi based on previous work employing imaging flow cytometry [15], are not circulating megakaryocytes that have also been reported in severe COVID-19 [[37], [38], [39]]. High circulating microthrombi and low αIIbβ3 activation in ICU patients are not conflicting observations since ultralarge von Willebrand factor [40,41] and fibrin [42] can support the formation of microthrombi in a αIIbβ3-independent manner. We speculate that the presence of circulating microthrombi negatively correlated with lung function because microthrombi could obstruct the lung microcirculation and limit gas exchange, in line with the numerous reports of pulmonary microthrombosis in postmortem histopathologic studies [3,43]. Despite the old age (Table) and the infection, the vaccinated patients did not display hyperactive platelets and circulating microthrombi and did not experience pulmonary complications.

In addition, vaccinated patients had a distinct pattern of PLAs. PLAs are not only useful biomarkers but also have important pathophysiological functions in modulating both innate and adaptive immune cells [44]. Formation of platelet–neutrophil aggregates during viral infections [2,45] promote neutrophil activation and NETs release [46]. Although NETs are deployed to capture invading pathogens, they are associated with poor outcome and severe ARDS in COVID-19 since they promote tissue damage and thrombosis [2,3]. We found high levels of platelet–granulocyte aggregates in all unvaccinated patients with COVID-19 (ICU and non-ICU) but not in the vaccinated patients with COVID-19. Platelet–granulocyte aggregates correlated with a reduced lung function, as if the vaccination could prevent the damaging effects of an excessive immunothrombotic response.

Conversely platelet–lymphocyte aggregates were very low in severe patients and elevated in patients with mild/moderate symptoms, both with and without vaccination, reflecting the importance of an effective adaptive immune response in controlling the disease. We have recently shown that in healthy adults, platelet–lymphocyte aggregates form shortly after vaccination and that increased levels of platelet–B cell aggregates 3 to 10 days after vaccination correlate with a faster antibody response [7,47]. The vaccinated patients had significantly higher levels of platelet–B cell aggregates compared to the ICU and the non-ICU unvaccinated patients. The platelet-bound B cells expressed higher levels of the activation marker CD69 and lower levels of L-selectin (CD62L), which is an early marker of antibody-secreting cell commitment during the first stages of B cell activation [17], suggesting that the platelet–B cell interaction could promote the antibody response. Consistently, previous studies in mice [48] and *in vitro* [49] demonstrate a role of platelets in positively regulating B cell differentiation and antibody production. Our laboratory is currently investigating the role of platelets in B cell modulation in health and disease, which will provide further insight into the meaning and significance of detecting platelet–B cell aggregates in patients.

Collectively these observations suggest that vaccinated subjects, by evoking a faster adaptive response to the virus, seem to be protected from an excessive innate immune response that concurs to tissue damage and drives aberrant platelet activation and formation of pathological platelet–platelet and platelet–neutrophil aggregates that cause the occlusion of the pulmonary microcirculation and exacerbate the severity of COVID-19. A recent longitudinal study comparing the cytokine response of vaccinated and unvaccinated patients with COVID-19 confirms that a preemptive vaccination affects not only the adaptive immune response, but it is also associated with a reduction of the inflammatory response during SARS-CoV-2 breakthrough infections [50].

Interestingly, incubation of a P-selectin inhibitor with whole blood from patients with COVID-19 significantly reduced the interactions of platelets with granulocytes and monocytes but not with lymphocytes, which express less P-selectin glycoprotein ligand-1. These data might suggest that P-selectin could be a promising pharmacological target to reduce the pathogenic effects of immunothrombosis while minimally affecting the adaptive immune response. However, 2 clinical trials testing the P-selectin inhibitor crizanlizumab failed to show efficacy in hospitalized patients with COVID-19 [18,19]. Since immunothrombosis is a physiological mechanism of host protection, more studies are needed to determine the right timing and setting in which this type of intervention would be beneficial.

We recognize that our study has several limitations. First, patient enrollment occurred across several months. Since we do not have consistent sequencing data on the SARS-CoV-2 variants infecting the enrolled patients, we could not exclude the confounding effect of the different variants. Second, since patients were hospitalized in different clinical wards, the only clinical data that we could consistently collect and correlate with the laboratory findings were D-dimer levels and the PaO_2_/FiO_2_ ratio as a measure of lung function. Third, we did not include in our study healthy vaccinated individuals because it was difficult to enroll healthy volunteers matched for age during the pandemic. However, we do not expect that the platelet changes that we observed previously in healthy volunteers after immunization [7] would persist long since they are part of the short-term innate immune response to the vaccine. Lastly, being observational our study does not provide causal evidence of a role of vaccination in protecting from platelet hyperactivation and thromboinflammatory complications. Despite these limitations, our study has the strength to compare markers of platelet activation and immunothrombosis with high granularity in vaccinated and unvaccinated patients with COVID-19. This approach will be useful to identify novel biomarkers and open new research avenues on the interplay between platelets and immunity.

In conclusion, our study provides evidence that vaccinated patients with COVID-19 are not associated with platelet hyperactivation and are characterized by PLAs that foster immune protection but not excessive immunothrombosis. These findings advocate for the importance of vaccinations in preventing severe COVID-19.
