# Supplementary material for: Breakthrough infections after COVID-19 vaccinations do not elicit platelet hyperactivation and are associated with high platelet–lymphocyte and low platelet–neutrophil aggregates
Source: Res Pract Thromb Haemost. 2023 Nov 14;7(8):102262. doi: 10.1016/j.rpth.2023.102262 (PMC10772876; doi:10.1016/j.rpth.2023.102262)
Supplement: Supplementary Figures S1–S3 [file mmc1.pdf]

Supplemental Figures

Figure S1. Gating strategy to identify leukocyte subsets and quantify platelet-leukocyte aggregates.

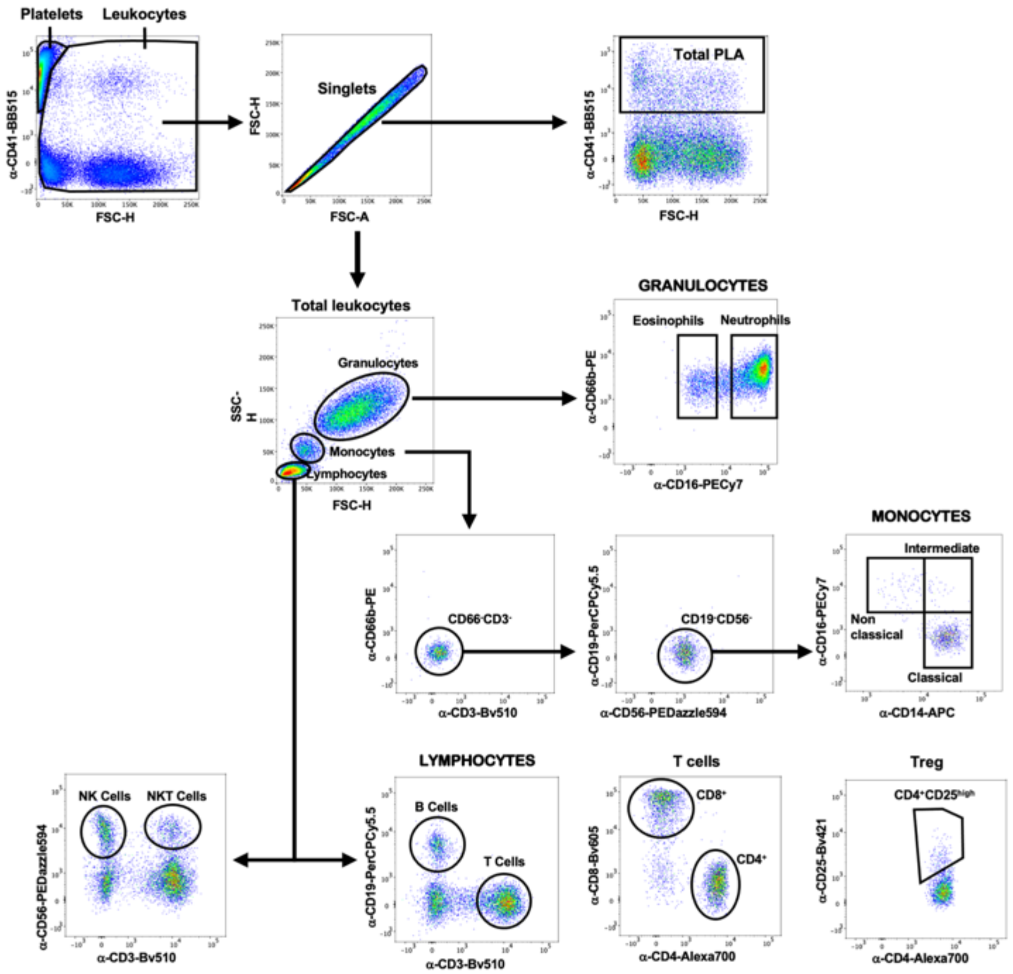

Upon staining and red blood cell lysis, single platelets were excluded based on forward side scatter (FSC-H) and CD41a^{+} staining. At least 50,000 events were acquired in the singlets gate to exclude doublets. Among the singlets, leukocyte subsets were first identified based on morphological parameters in the FSC-H/SSC-H plot. Among the granulocytes, neutrophils (CD66^{+}CD16^{+}) and eosinophils (CD66^{+}CD16^{-}) were identified. Among the monocytes, CD3^{+}CD66^{+}CD19^{+}CD56^{+} events were excluded, and classical (CD14^{+}C16^{-}), non-classical (CD14^{dim}CD16^{+}) and intermediate monocytes (CD14+CD16+) were identified. Among lymphocytes, CD4^{+} (CD3^{+}CD4^{+}), CD8^{+} (CD3^{+}CD8^{+}) and regulatory (CD3^{+}CD4^{+}CD25^{high}) T cells, natural killer (NK, CD3^{-}CD56^{+}), natural killer T-cells (NKT, CD56^{+}CD3^{+}) and B-cells (CD3^{-}CD19^{+}) were identified. Platelet-leukocyte aggregates (PLA) were identified based on the expression of CD41a among total leukocytes and in the individual leukocyte subpopulations. Fluorescence-minus-one controls were performed to ensure proper gating.

Figure S2. Gating strategy to identify platelet-platelet aggregates (microthrombi) in whole blood.

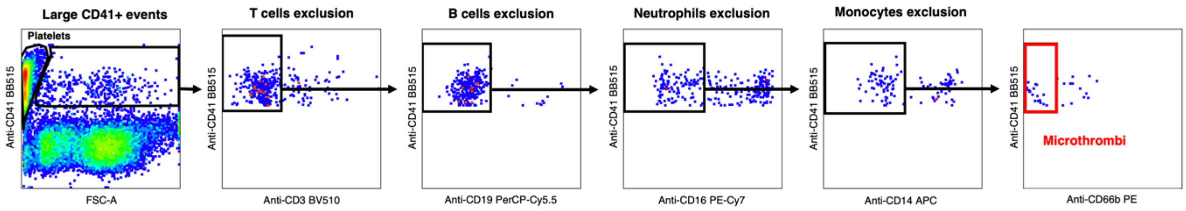

Circulating platelet-platelet aggregates (microthrombi) were identified by gating for CD41a+ events larger (FSC^{high}) than individual platelets and then by excluding events positive for CD3 (T-cells), CD19 (B-cells), CD16, CD66b (neutrophils) and CD14 (monocytes). Flow cytometry acquisition was performed on a BD LSR Fortessa and data analysed with the FlowJo LLC software.

Figure S3. Impaired responsiveness of ICU COVID-19 patients in the presence or absence of plasma.

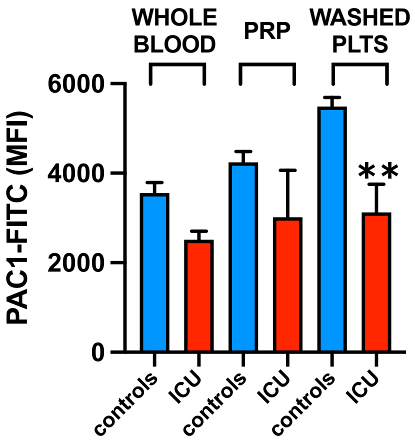

Convulxin-stimulated integrin activation (measured by flow cytometry via PAC-1 binding) of platelets of control (blue) or COVID-19 from ICU (red) patients in diluted whole blood, platelet-rich plasma (PRP) or resuspended in Tyrode’s buffer (washed platelets).
